# Genetic diversity and transmissibility of imported *Plasmodium vivax* in Qatar and three countries of origin

**DOI:** 10.1038/s41598-018-27229-z

**Published:** 2018-06-11

**Authors:** Mohammed H. Abdelraheem, Devendra Bansal, Mohammed A. Idris, Moawia M. Mukhtar, Muzamil M. Abdel Hamid, Zainb S. Imam, Sisay Getachew, Rakesh Sehgal, Hargobinder Kaur, Amal H. Gadalla, Salam Al-Hamidhi, Zainab Al-Hashami, Ali Al-Jabri, Ali A. Sultan, Hamza A. Babiker

**Affiliations:** 1 Department of Microbiology and Immunology, College of Medicine and Health Sciences, Sultan Qaboos University, Muscat, Oman; 2 Department of Microbiology and Immunology, Weill Cornell Medicine-Qatar, Cornell University, Qatar Foundation-Education City, Doha, Qatar; 3 Institute of Endemic Diseases, University of Khartoum, Khartoum, Sudan; 4 Addis Ababa University, Addis Ababa, Ethiopia; 5 Department of Medical Parasitology, Postgraduate Institute of Medical Education and Research, Chandigarh, India; 6 Division of Population Medicine, School of Medicine, College of Biomedical Sciences, Cardiff University, Cardiff, UK; 7 Department of Biochemistry, Faculty of Medicine and Health Sciences, Sultan Qaboos University, Muscat, Oman; 8 Institute of Immunology and Infection Research, School of Biological Sciences, Ashworth Laboratories, University of Edinburgh, Edinburgh, UK

## Abstract

Malaria control program in the Arabian Peninsula, backed by adequate logistical support, has interrupted transmission with exception of limited sites in Saudi Arabia and sporadic outbreaks in Oman. However, sustained influx of imported malaria represents a direct threat to the above success. Here we examined the extent of genetic diversity among imported *P. vivax* in Qatar, and its ability to produce gametocytes, compared to parasites in main sites of imported cases, the Indian subcontinent (india) and East Africa (Sudan and Ethiopia). High diversity was seen among imported *P. vivax* in Qatar, comparable to parasites in the Indian subcontinent and East Africa. Limited genetic differentiation was seen among imported *P. vivax*, which overlapped with parasites in India, but differentiated from that in Sudan and Ethiopia. Parasite density among imported cases, ranged widely between 26.25–7985934.1 *Pv18S* rRNA copies/µl blood, with a high prevalence of infections carried gametocytes detectable by qRT-PCR. Parasitaemia was a stronger predictor for *P*. *vivax* gametocytes density **(**r = 0.211, P = 0.04). The extensive diversity of imported *P. vivax* and its ability to produce gametocytes represent a major threat for re-introduction of malaria in Qatar. The genetic relatedness between *P. vivax* reported in Qatar and those in India suggest that elimination strategy should target flow and dispersal of imported malaria into the region.

## Introduction

Global concerted and collaborative efforts, backed by WHO, have resulted in remarkable reduction in the burden of malaria, and disease elimination in many areas. Nonetheless, imported malaria represents a significant threat to the above achievements^1^. The escalating volume of international travel among people in endemic areas, driven by desire to improve quality of life and political unrest, has increased the risk of malaria importation into non-endemic areas, including Europe^2^.

Local malaria transmission in the Gulf Cooperation Council (GCC) countries has been interrupted as a result of sustained vector control^3^, and active case detection to define existing malaria foci. Consequently, this has brought local transmission to a halt in many countries in the region, including Bahrain, Kuwait and the United Arab Emirates (UAE), and more recently Oman^3^. However, some sites in Yemen and southern Saudi Arabia remain malarious, with a high prevalence of drug-resistant *Plasmodium falciparum*^4^. The success of control programs in the region has prompted the health authorities in GCC countries to shift strategies towards malaria elimination and prevention of re-introduction in receptive areas^5^.

However, the above success is vulnerable to a possible resurgence of local transmission caused by imported parasites via asymptomatic travellers from malaria-endemic areas^6,7^. The risk of malaria resurgence depends on the combined effect of receptivity and vulnerability. Receptivity is a function of the presence of local vectors and environmental conditions that favour malaria transmission, while vulnerability reflects the probability of importation of malaria parasites into a country^8^. The region is highly vulnerable to re-introduction of malaria, given the high volume of immigration from malaria endemic regions. This has been implicated in the persistence of high rates of imported malaria in many GCC countries^9^. For example, while a significant reduction in locally acquired malaria cases was observed between 2000 and 2014 in Saudi Arabia, imported cases remained consistent averaging between 250 and 830 cases per year^6,10^. Imported malaria is also common in Qatar, Bahrain, Kuwait and the UAE^7,11–13^, and has been implicated in repeated malaria outbreaks in Oman^14^. In Qatar, malaria transmission has been interrupted since 1970^15^, however, a constant influx of imported malaria by the large number of immigrant workers from the Indian Subcontinent, and Sub-Saharan Africa, poses a risk for re-introduction^11,16^. Receptivity of the country to a re-introduction of malaria is evident by the presence of two potential vectors, *Anopheles stephensi* and *A. multicolour*^17^. The proportion of all imported malaria reported amongst travellers in Qatar has increased over the past decade, with *P. vivax* being the main prevalent parasite^11^.

The impact of imported malaria, often as asymptomatic infection, on local epidemiology and local transmission is not well defined in Qatar and surrounding regions. However the ability of asymptomatic *P. falciparum* and *P. vivax* infections to produce gametocytes is well documented^18,19^. Currently there is no clear information on the actual magnitude of asymptomatic parasite reservoir in areas where malaria has been eliminated in the GCC countries and its population structure. Thus, the present study examined the source of imported *P. vivax* into the transmission free setting of Qatar, assessed its genetic complexity and its ability to produce gametocytes and transmit malaria. In addition, we compared the extent of diversity and genetic relatedness of imported parasites into Qatar with that in three countries of origin of immigrant malaria into Qatar, in Africa (Sudan and Ethiopia) and the Indian Subcontinent (India). Such knowledge would allow regional and national control programs to develop targeted policies to reduce the size of infectious reservoir, define the source of outbreaks and limit the risk of re-introduction of malaria.

## Results

### Demographic characteristics of imported malaria in Qatar

**Figure 1:**
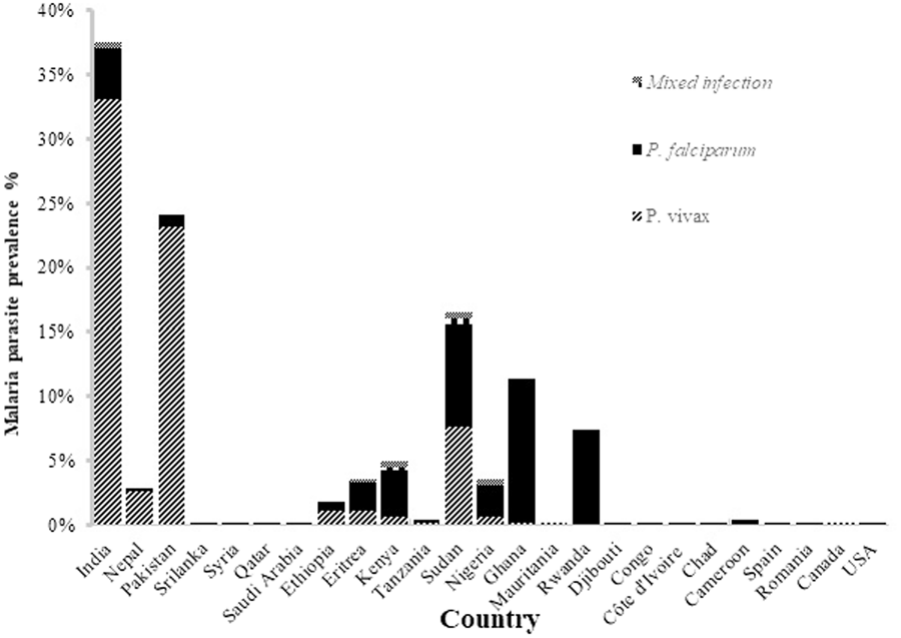
Prevalence of *P. vivax*, *P. falciparum* and mixed species infection among imported malaria cases in Qatar between September 2013 and October 2016.

Out of 583 patients examined by microscopy for malaria between January 2013 and October 2016 in Qatar, 448 (76.8%) were found to be positive. Species-specific PCR revealed a high prevalence of *P. vivax* cases (n = 318 [70.9%]), while *P. falciparum* (n = 118 [26.3%]) and mixed infection, with *P. falciparum* and *P. vivax,* was less frequent (n = 12 [2.7%]) (Fig. 1).

The vast majority of imported *P. vivax* cases were young males (93.7%), mean age 32 years, from the Indian Subcontinent (n = 264 [83%]); including, India (n = 148 [46.5%]), Pakistan (n = 104, [32.7%]) and Nepal (n = 12, [3.7%]). However, a small proportion of imported *P. vivax* cases were from Africa (n = 53 [16.7%]), including Sudan (n = 24, [7.5%]), and other African countries (n = 19, [6%]). On the contrary, the vast majority of imported *P. falciparum* cases were from Africa (n = 88 out of 118, [75%] [75%]), with a fewer number of patients from the Indian Subcontinent (n = 23, [19%] [%]) and others (n = 7 [6%]) (Table 1 and Fig. 1).

**Table 1:** Characteristics of 265 imported *P. vivax* cases reported into Hamad General Hospital, Qatar, between September 2013 and November 2016.

Nationality	Number of cases n (%)	Mean age Years	Gender Male	Average parasitaemia
Indian	132 (49.81%)	30	126 (97%)	0.62%
Pakistani	89 (33.58%)	33	83 (92%)	0.70%
Nepalese	10 (3.77%)	27	10 (100%)	0.45%
Sudanese	24 (9.05%)	39	19 (76%)	0.58%
Eritrean	4 (1.5%)	48	4 (100%)	1.35
Ethiopian	4 (1.5%)	28	4 (100%)	0.85
Kenyan	1 (0.37%)	25	1 (100%)	0.3%
Canadian	1 (0.37%)	39	—	0.7%

### Parasitaemia and gametocyte carriage among imported *P. vivax*

Ninety-five imported *P. vivax* isolates originated from the Indian Subcontinent (India, n = 59 [62.10%], Pakistan, n = 35 [36.84%] and Nepal, n = 1 [1.05%]) were successfully examined by qPCR and qRT-PCR to quantify total parasitaemia and gametocyte density, respectively. Total parasite density estimated as transcript of *Pv18s rRNA* ranged between 26.2 and 7985934.1 copies/µl of blood (median = 1029.0).

Out of 95 *P. vivax* isolates examined by RT-qPCR, 94 (98.9%) had detectable transcripts of *Pvs25*, indicative of the presence of gametocytes. The copy number of *Pvs25* transcript, among the examined isolates ranged from 0.33 to −78441.24 copies of *Pvs25*/µl of blood (median = 300.3).

**Figure 2:**
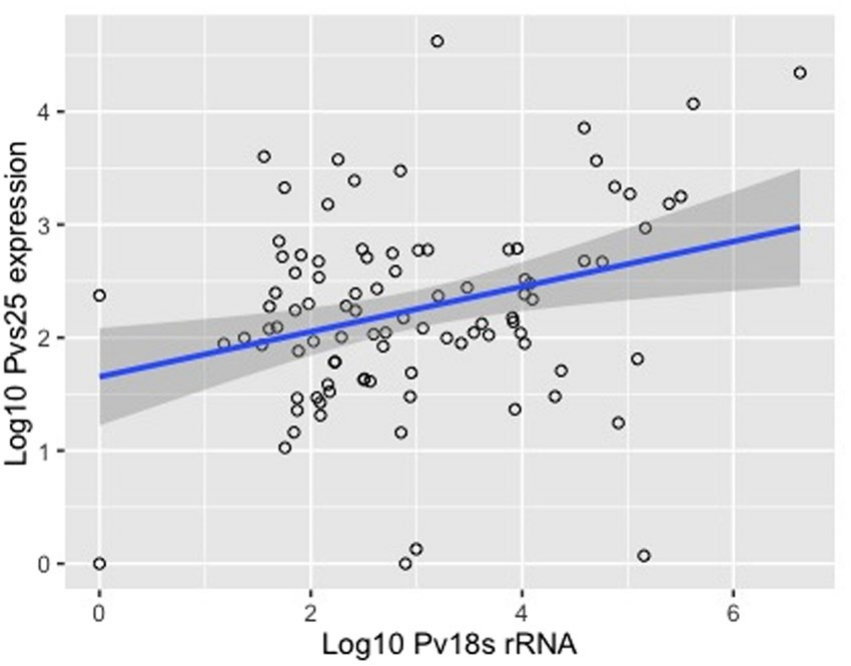
Association between parasite density and gametocyte density among imported *P. vivax* cases in Qatar (Correlation Coefficient = 0.211, P = 0.04).

Spearman’s rank-order correlation test showed a positive relationship between the copy numbers of *Pv18srRNA* and *Pvs25* transcripts (Correlation Coefficient, r = 0.211, P = 0.04), suggesting an association between parasitaemia and gametocyte density (Fig. 2).

### Diversity of *P. vivax*

Microsatellite analysis was carried out among 265 imported *P. vivax* isolates in Qatar, as well as *P. vivax* isolates collected in endemic sites, where the majority of imported malaria into Qatar originated, in Sudan (n = 137), Ethiopia (n = 87) and India (n = 40). There was a high degree of polymorphism in all microsatellites, with number of alleles per locus varied from 8 for MS1 to 38 for MS8 among imported parasites in Qatar, 6 for MS7 to 22 for MS20 among parasites in Sudan, 4 for MS7 to 22 for MS8 among parasites in Ethiopia and 6 for MS5 to 19 for MS8 among Indian isolates (Table 2).

**Table 2:** Number of isolates (N), alleles (Na), private alleles (PA) and estimated heterozygosity (H_E_) among imported *P. vivax* in Qatar and local parasites in Sudan, Ethiopia and India.

Locus	Imported cases (n = 265)				Sudan (n = 137)				Ethiopia (n = 87)				India (n= 40)			
	N	Na	PA	*H*_E_	N	Na	PA	*H*_E_	N	Na	PA	*H*_E_	N	Na	PA	*H*_E_
MS1	264	8	0	0.792	129	10	0	0.673	72	8	0	0.848	39	6	0	0.771
MS5	264	8	3	0.705	130	8	3	0.761	79	9	6	0.757	40	6	2	0.549
MS6	264	13	6	0.858	127	7	4	0.516	81	6	4	0.573	40	8	2	0.840
MS7	265	9	7	0.9	132	6	2	0.593	78	4	2	0.294	36	7	2	0.724
MS8	264	38	11	0.915	127	19	2	0.874	80	22	1	0.927	40	19	9	0.948
MS9	261	16	8	0.872	135	7	7	0.770	82	8	12	0.606	38	10	3	0.830
MS12	265	14	6	0.845	131	7	9	0.714	80	9	2	0.739	36	10	4	0.855
MS15	265	15	4	0.733	127	10	3	0.721	79	12	4	0.783	39	8	1	0.840
MS20	264	21	3	0.716	124	22	3	0.915	83	15	3	0.629	40	13	7	0.816
Average	264	15.7	5.8	0.782	129	10.6	3.6	0.726	79	10	4.2	0.684	39	9.6	3.3	0.797

There was an extensive diversity among imported *P. vivax* in Qatar (*H*_E_ = 0.782) similar to that seen in endemic sites in Sudan (*H*_E_ = 0.726), Ethiopia (*H*_E_ = 0.684) and India (*H*_E_ = 0.797) (Table 2). A total of 142 alleles in all loci were found among imported *P. vivax* in Qatar compared to 96, 93 and 87 alleles in Sudan, Ethiopia and India, respectively. However, many private alleles were seen in some loci in all sites, imported parasites, Qatar (n = 48), Ethiopia (n = 34), Sudan (n = 33) and India (n = 30) (Table 2).

The predominant allele at each locus in each isolate was used to construct multi-locus genotypes (haplotypes). Each *P. vivax* isolate, among imported cases as well as parasites collected in Sudan, Ethiopia and India, carried a distinct multi-locus genotype, with exception of 5 pairs (10 isolates) in Sudan each shared a distinct genotype.

### Linkage disequilibrium

The multi-locus genotype (haplotypes) data was used to estimate the standard index of association (I^S^_A_). This determines whether there is an association between alleles on different loci, and if the above high levels of diversity observed in *P. vivax* populations could be explained by frequent genetic exchange.

Limited linkage disequilibrium (LD) was seen among the imported cases in Qatar I^S^_A_ = 0.0050 while a relatively higher LD value was seen among parasites in Sudan (0.0958) compared to parasites from Ethiopia (0.0367) and India (0.0265) (Table 3). These values, suggest that a particular combination of alleles on different chromosomes exist at a higher frequency than that expected among randomly mating parasites.

**Table 3:** Analysis of linkage disequilibrium (LD) for 9 microsatellites among imported *P. vivax* in Qatar and local parasites in Sudan, Ethiopia and India.

Population	I^S^_A_	V_D_	V_e_	L_MC_	Status
Imported cases (Qatar)	0.0050	1.5096	1.4510	1.5047	LD
Sudan	0.0958	2.7666	1.5661	1.6705	LD
Ethiopia	0.0367	1.5336	1.1858	1.2044	LD
India	0.0265	1.4071	1.1611	1.2630	LD

I^S^_A_: Standardized Index of Association, V_D_: Observed mismatch variance, V_e_: Expected mismatch variance, L_MC_: Simulated 5% critical value.

### Population differentiation

To assess the genetic relatedness among imported *P. vivax* in Qatar, the isolates were divided into three populations (India [n = 132], Pakistan [n = 89] and Sudan [n = 24]) based on the nationality of patients. Pairwise analysis showed a close relationship between parasites from Pakistan and India (*F*_ST_ = 0.0008), compared to that seen between parasites from Sudan and India (*F*_ST_ = 0.0037) or Sudan and Pakistan (*F*_ST_ = 0.0011). Nonetheless, imported *P. vivax* from the three countries can be considered as one population. Similar close genetic relatedness was seen between imported *P. vivax* in Qatar, treated as one population, and isolates collected in India (*F*_ST_ = 0.0594). However, imported *P. vivax* in Qatar was slightly diverged from local parasite in Sudan (*F*_ST_ = 0.1435) and Ethiopia (*F*_ST_ = 0.1896).

**Figure 3:**
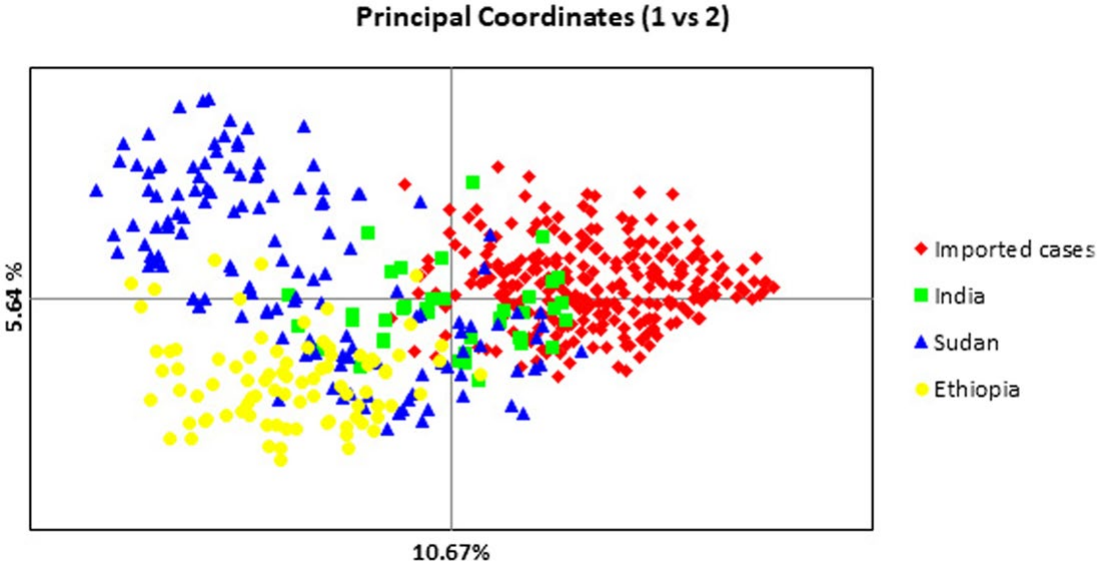
Principal Co-ordinates analysis (PcoA) of *P. vivax* among imported cases in Qatar and those from endemic sites in India, Sudan and Ethiopia. The amount of variation explained by each axis is shown as a percentage of the overall variation.

The above results were supported by Principal Component Analysis (PCoA) (Fig. 3). The Ethiopian *P. vivax* population was distanced from the imported parasites in Qatar, while the Sudanese and Ethiopian parasites were slightly overlapped (Fig. 3). Analysis of molecular variance (AMOVA) indicated that most of the genetic variation (86%) was contained within parasites in each region and only 14% can be explained by differences between regions.

**Figure 4:**
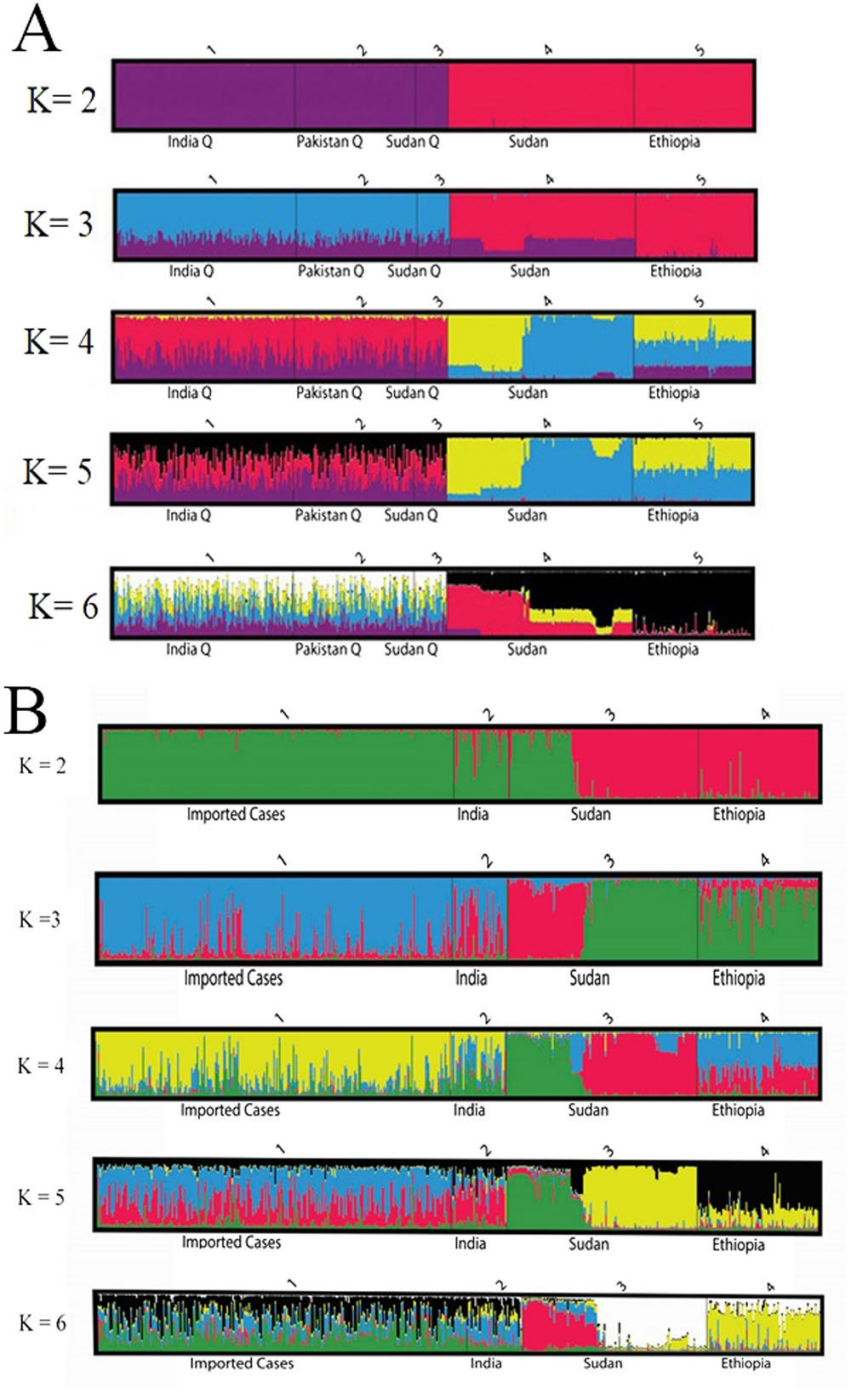
(**A**) Population Structure. Bar plot illustrating the population structure at K = 2–6 in *P. vivax* among imported cases in Qatar and parasites from endemic sites in India, Sudan and Ethiopia. Each vertical bar represents an individual sample, and each colour represents one of the K clusters (subpopulations). (**B**) Population Structure. Bar plot illustrating the population structure at K = 2–6 in *P. vivax* among imported cases in Qatar and parasites from endemic sites in India, Sudan and Ethiopia. Each vertical bar represents an individual sample, and each colour represents one of the K clusters (subpopulations).

Structure analysis showed a clear separation between the imported *P. vivax* in Qatar and the East African isolates (Sudan and Ethiopia) when K = 2. However, when K = 3 there was some overlap between Sudanese isolates and imported cases in Qatar in agreement with the *F*_ST_ analysis. However, when K = 4, a clear overlap between the imported parasites to Qatar and parasite in Sudan and Ethiopia (Fig. 4A), suggesting a likely existence of 4 sub-populations (Fig. 4A). Moreover, re-analysis of the data using the imported cases as single population revealed a high relationship between the isolates collected in India with the imported *P. vivax* in Qatar compared to the parasites in Sudan and Ethiopia (Fig. 4B).

### Multiplicity of infection (MOI)

MOI defined as presence of more than one allele in any locus, was high among imported *P. vivax* from India (67.9%), Sudan (60.6%), and Pakistan (50.5%). Similar, high MOI was seen among local *P. vivax* in Sudan (44.70%), Ethiopia (67%) and India (62.5%).

Moreover, the minimum number of clones (MNC), estimated as the minimum number of alleles per locus, across all loci, was relatively low among imported *P. vivax* from India (1.6), Sudan (0.7), and Pakistan (1.2) compared to that seen among endemic parasites in Sudan (2.4), Ethiopia (2.00) and India (2.00).

## Discussion

The GCC countries have achieved great success in the fight against malaria. Four out of the six states accomplished complete interruption of malaria transmission and a malaria-free status, while limited sites in southwest Saudi Arabia^10^ and sporadic outbreaks in Oman^14^ represent the final stage in the battle to eliminate endemic malaria in the region. However, the above success is threatened by the high influx of imported malaria by young immigrants from endemic countries in the Indian subcontinent (India, Pakistan) and East Africa (Sudan).

The findings of the present study are in line with previous reports, demonstrating that the Indian Subcontinent is the most likely source for imported *P. vivax* malaria into GCC countries while Africa is the main origin of *P*. *falciparum* malaria. For some decades, the oil-rich region has been a popular destination for temporary labour workers, seeking employment opportunities. The high migratory turnover of immigrants from malarious areas is accompanied by increased reports of imported malaria in GCC countries^9^, reflecting a high vulnerability for re-introduction of malaria in areas where the disease has been eliminated. Though, receptivity, defined as the presence of the *Anopheles* vector, is limited as the result of current efficient vector control programs^8,20^. Nonetheless, autochthonous malaria cases were seen near livestock farms and building sites, where a large number of immigrants work, and environmental conditions favour vector habitats^14^. Analysis of a recent malaria outbreak in Oman identified *Anopheles culicifacies* as the vector involved^14^. Therefore, to minimize vulnerability, to re-introduction of malaria, a stringent surveillance system, based on highly sensitive molecular tools, should be structured to ensure timely detection and management of outbreaks seeded by imported malaria.

The vulnerability to re-introduction of local transmission is emphasised by the high prevalence of gametocytes carriage among imported *P. vivax* in Qatar. Out of 95 *P. vivax* isolates successfully examined by RT-PCR, 83 (87.37%) harboured gametocytes evident by detectable transcripts of *Pvs25* gene. This accords well with the findings of the high prevalence of low-density gametocyte carriage among *P. vivax* infection in many endemic sites^21^. *P. vivax* isolates examined in the present study were obtained from clinical cases. However, imported malaria often exists as asymptomatic low-level parasitaemia detectable only by PCR^22^ that may easily be missed by traditional microscopy and serological surveillance methods. An additional feature of asymptomatic infection, of particular relevance to prevention of re-introduction in areas where malaria has been eliminated, is its extended longevity. Asymptomatic *P. falciparum* infection can persist for several years, and discovered only when an individual develops clinical malaria^23^. Clinical presentations of *P. falciparum* malaria among immigrants have been found to delay by as long as 8 years after patients have left malaria-endemic areas^24^. Thus, asymptomatic parasite carriers can sustain long-term threat to malaria elimination programs.

The presence of gametocyte among low-density asymptomatic parasite carriers is well documented^25^. Amplification-based techniques are sensitive enough to detect and quantify gametocytes as low as 0.02–10 gametocytes per microliter^25,26^. Such low gametocytes densities are unlikely to be detected by microscopy and can make significant contributions to transmission^23,27^. In addition, parasites can increase gametocyte conversion rate in response to clues for transmission opportunity^25,27,28^. For example, the reappearance of *Anopheles* mosquitoes following the long dry period in areas of seasonal transmission can promote surge in gametocyte densities among asymptomatic parasite carriers^26^. This may explain the success of imported asymptomatic malaria to raise regular outbreaks in receptive sites, with low mosquito density in some GCC countries such as Oman^14^ and southern Europe^29^. Thus, molecular surveillance of migrants from endemic areas, in GCC countries and other receptive areas, can estimate the risk of re-introduction and allow targeted control measures to interrupt potential transmission.

High level of diversity was seen among imported *P. vivax* parasites into Qatar [average *H*_E_ = 0.782], similar to that in endemic sites where the majority of imported cases originate, in India [average *H*_E_ = 0.797] and East Africa (Sudan [average *H*_E_ = 0.726], Ethiopia [average *H*_E_ = 0.684]). This is consistent with previous reports on diversity of *P. vivax* in the Indian Subcontinent^30^ and East Africa^31^. The high level of diversity seen among imported *P. vivax* in Qatar reflects a constant flow of importation of diverse strains from multiple origins. This hypothesis is supported by the close genetic relatedness of imported *P. vivax* in Qatar and local parasite in India as shown by *F*_ST_ and PCoA analysis (Fig. 3), and some overlap with parasites in East Africa. The introduction of novel lineages into the region can increase effective population size (*Ne*) and enhance the parasite diversity, as there is a direct relationship between the expected level of diversity and *Ne*^32^. In addition, the appearance of drug resistance in countries of origin such as India^33^, Pakistan^34^ and Ethiopia^35^ can augment the ability of the parasite to establish and evolve in the face of current control measures, if local transmission arises.

The extent of diversity among imported *P. vivax* is higher than that seen amongst *P. falciparum* reported by immigrants in Qatar at the same time of the present study^36^. This is in line with data from different endemic areas that examined sympatric *P. vivax* and *P. falciparum* populations^37^. This has been attributed to the fact that the global *P. vivax* population is older, efficient in transmission, very diverse and less structured than *P. falciparum*^37,38^. This is conistsnt with the findings of low level of genetic differentiation between imported *P. vivax* in Qatar and local parasites in India (*F*_ST_ = 0.0594), and moderate differentiation between imported parasites in Qatar and that in East Africa (Fig. 3). Thus, analysis of imported *P. vivax* in transmission-free areas and autochthonous malaria in the GCC countries, can predict the genetic relatedness and the likelihood of success of integrated intervention strategy applied across the whole region.

In summary, the present study highlighted the vulnerability of GCC countries to resurgence of malaria via parasite carried by immigrants from endemic areas. Imported *P. vivax* infection in Qatar is characterised by a high rate of genetic diversity and ability to produce gametocytes to transmit disease. The genetic diversity and complexity of imported parasites can lead to appearance of novel genotypes, that can escape current treatment regimen should local transmission starts. However, the risk of receptivity and malaria resurgence is limited, as a result of adequate vector control programs, despite the presence of local vectors and ecological conditions favourable to malaria transmission^39^.

## Material and Methods

### Study sites and *P. vivax* isolates

A total of 583 patients reported to Hamad Medical Corporation (HMC) and Alkhor Hospital; Hamad General Hospital in Al-Doha city and Alkhor Hospital in the north area are the two main hospitals within HMC that assigned to admit patients with malaria, were examined microscopically for malaria between January 2013 and October 2016. All cases were diagnosed using conventional microscopic examination of Giemsa stained thick (100 fields) and thin blood (1000 RBCs). A total of 448 (76.8%) were found to be positive for the malaria parasites.

All subjects were interviewed using a structured questionnaire to collect demographic information, including age, gender, nationality, history of travel and treatment. Patients were given treatment as per the Ministry of Public Health, Qatar.

The present study focused on analysis of 265 isolates obtained from patients with *P. vivax* infection, in Qatar. In addition, we also used 137 confirmed infected samples from Sudan, collected in Whatman qualitative filter paper, grade 3 between 2013–2014, 87 confirmed infected samples from Ethiopia collected in Whatman qualitative filter paper, grade 3 between 2010–2013 and 40 confirmed *P. vivax* samples from India collected between 2014–2016.

Ethical approvals were obtained from the Institutional Review Board of WCM-Q and HMC (Protocol no. 14-00097), ethical committee of the Postgraduate Institute of Medical Education and Research, Chandigarh, India (PGI/IEC/2014/88), Research Review Committee of Institute of Endemic Diseases, University of Khartoum (certificate research number 9/2016), and the Ethiopian isolates were reviewed and approved by the respective Ethical Boards of the Addis Ababa University College of Natural Sciences, Ethiopia (RERC/002/05/2013) and National Research Ethics Review Committee of Ethiopia (Ref.no. 3.10/580/06)^35^. Samples were collected after obtaining a written informed consent from the patients or guardians. All experiments in this study were performed in accordance with relevant guidelines and regulations of the above institutes.

### Extraction of DNA and RNA

DNA was extracted from 200 µl of venous blood from the imported *P. vivax* isolates in Qatar and parasites collected in India, using QIAamp DNA Blood Mini Kit as described by the manufacturer (QIAGEN,CA, USA). DNA was isolated from blood collected on filter paper (Whatman qualitative filter paper, grade 3) from Sudan and Ethiopia using Chelex extraction method^40^.

RNA was extracted from 100 µl blood of *P. vivax* isolate obtained from imported cases in Qatar using SV Total RNA Isolation kit (Promega, UK). Conventional PCR was used to confirm the absence of co-extracted genomic DNA, and then RNA samples were converted to cDNA using the High Capacity cDNA Reverse Transcription Kit (ThermoFisher, UK).

### Detection and quantification of total parasitaemia and gametocytes among imported *P. vivax* in Qatar

Species identification of *P. vivax* was confirmed using species-specific PCR as described by^41^. Total parasitaemia was quantified as copy number of the *18S rRNA* transcripts in reverse transcriptase quantitative polymerase chain reaction (RT-qPCR) assays based on TaqMan probe chemistry, primers and probes sequences are provided in Table 4^19^. *Pv18s rRNA* copy numbers were estimated using in-plate standard curve generated from a 10-fold serial dilution (starting with 3.79 × 10^7^ copies/µl, limit of detection 0.37 copy/µl) of a purified PCR product. Gametocytes were detected and quantified using RT-qPCR, targeting transcripts of *Pvs25* gene, as described elsewhere^21^, with the use of modified probes and primers sequences (Table 4).

**Table 4:** Primers and probes used for qRT-PCR of *Pv18s rRNA* and *Pvs25*.

Name	Sequence (5′-3′)
PV18s-qFW	TCT AGC TTA ATC CAC ATA ACT GAT AC
PV18s-qRV	CCR AAG CAA AGA AAG TCC TT
VIV 18s-probe	6-Fam- TCG TAT CGA CTT TGT GCG CAT TTT GCT-BHQ-1
Pvs25-qFW	AAG TGT AYG TGT AAC GAA GGG
Pvs25-qRv	TAT ACA CTG GCC AAA TTC CC
Pvs25-qPr	6-Fam-CGC ATG CTT TGC CTA GGG TTT CTT TCT- Tamra

### Genotyping of *P. vivax*

We examined 9 polymorphic single copy tri-nucleotide repeats microsatellites (MS1, MS5, MS6, MS7, MS8, MS9, MS12, MS15, MS20), distributed across the whole parasite genome. In 25 μl premix containing Taq polymerase (2U), MgCl_2_ (1.5 mM), dNTPs (50 µM of each dNTPs) and 10 pmole of each primer. PCR conditions were, 94 °C/3 min, 35 cycles at 94 °C/30 s, 59.8 °C/30 s and 72 °C/45 s, and a final extension step at 72 °C/5 min^42^.

PCR products were analysed using ABI 3130XL Genetic analyser (Applied Biosystems,UK) with relative to an internal reference Genescan 500 LIZ (Applied Biosystems, UK). GeneMapper v4.0 (Applied Biosystems, UK) for allele calling and quantification of peak height^43^.

### Data analysis

#### Multiplicity of Infection (MOI)

The multiplicity of infection (MOI) refers to presence of more than one allele per isolate at least in one locus. To avoid overestimation of MOI, multiple alleles per locus were scored if electrophoretic peaks corresponding to minor alleles were ≥33% the height of the predominant allele^44^. The minimum number of alleles across the 9 loci in each sample was calculated and this index value represented the minimum number of clones (MNC) (genotypes) per isolate. Then the average MNC for all isolates in each region was calculated^44^.

### Population genetic indices

Genetic diversity parameters were calculated for the entire dataset using GenAlex v6.5^45^. This included determining the number of alleles per locus, and expected heterozygosity (*H*_E_). These two parameters were used to assess the level of polymorphism at each locus and determine diversity. *H*_E_ was calculated using the formula for ‘unbiased heterozygosity’ also termed haploid genetic diversity, *H*_E_ = [n/(n − 1)][1 − ∑p^2^] where *n* is the number of isolates and *p* the frequency of each different allele at a locus^46^.

The predominant allele in each of the examined 9 microsatellites was used to construct multi-locus haplotypes to determine whether the *P. vivax* populations in different regions comprised a single panmictic population with a high degree of genetic exchange. Multilocus linkage disequilibrium (LD = non-random association of allele among loci) of the alleles at pairs of loci was estimated as the standard index of association (I^S^_A_) using the program LIAN version 3.5^47^. The software tests for independent assortment of alleles by determining the number of loci at which each pair of MLGs differs, and from the distribution of mismatch values a variance V_D_ (the variance of the number of alleles shared between all pairs of haplotypes observed in the population) is calculated, which is then compared with the variance expected for LE, termed V_e_. The null hypothesis that V_D_ = V_e_ is tested by a Monte Carlo simulation and a parametric method and the results provide 95% confidence limits, which are denoted L_MC_ and L_PARA_, respectively. If there is limited or no association between alleles at different loci, indicating panmixia, a value close to zero is obtained, whereas if association is detected, a value significantly greater than 0 is obtained, indicating non-panmixia^47^. The variance of pairwise difference (V_D_) between the data and that predicated for panmixia^14^ and L (L_MC_ & L_PARA_), were calculated in order to test the hypothesis of panmixia. Therefore, when the V_D_ value exceeds the L value, LD is indicted and the null hypothesis of panmixia is discarded. When the V_D_ is less than L, LE is indicated and the null hypothesis of panmixia is accepted^47^.

The genetic association between population pairs was evaluated by Wright’s fixation index (*F*_ST_) using Gene pop web interface^48^. Molecular variance (AMOVA) was calculated to estimate the variation within and between population and Principal component analysis (PCoA) was used to visualise the relationship between MLGs using GenAlEx 6.5^45^.

Structure software was used to elucidate the genetic structure and to detect the most likely number of clusters (K)^49^. STRUCTURE analysis runs were performed exploring K from 1 to 6 (10 iterations each), consisting of a burn-in period of 10,000 iterations followed by 100,000 Markov Chain Monte Carlo (MCMC) iterations, assuming a mixture model and correlated allele frequencies. K was defined according to^49^ by calculating the rate of change of *K*, *ΔK* using STRUCTURE HARVESTER v0.6.94^50^. Additional analysis of structure results was conducted using CLUMMP^51^ and DISTRUCT^52^. CLUMMP aligns the cluster assignment across replicate analyses while DISTRUCT performs a graphical display of the aligned cluster assignments.
